# *Parabacteroides distasonis* alleviates *Clostridioides difficile* infection in mice while modulating secondary bile acids

**DOI:** 10.1080/21505594.2026.2697574

**Published:** 2026-07-05

**Authors:** Min Zhao, Baojiang Wen, Zirou Ouyang, Jing Yang, Huimin Yang, Jiafeng Zhao, Cuixin Qiang, Weigang Wang, Pu Qin, Yanan Niu, Jianhong Zhao

**Affiliations:** a Hebei Provincial Center for Clinical Laboratories, The Second Hospital of Hebei Medical University, Shijiazhuang, Hebei, China; b Department of Laboratory, Tianjin Medical University Cancer Institute and Hospital, Tianjin Key Laboratory of Digestive Cancer, National Clinical Research Center for Cancer, Key Laboratory of Cancer Prevention and Therapy, Tianjin’s Clinical Research Center for Cancer, Tianjin, China; c Department of Clinical Laboratory Center, Shaoxing People’s Hospital, Shaoxing, Zhejiang, China; d Hebei Technology Innovation Center for Precision Medicine Diagnosis and Quality Control, Shijiazhuang, Hebei, China; e Hebei Engineering Research Center of Medical Microbiome and Clinical Translation, Shijiazhuang, Hebei, China

**Keywords:** *Clostridioides difficile*, *Clostridioides difficile* infection, *Parabacteroides distasonis*, gut microbiota, bile acid

## Abstract

*Clostridioides difficile* infection (CDI) is a major cause of antibiotic-associated diarrhea, with frequent recurrences closely linked to antibiotic-induced dysbiosis of the gut microbiota and bile acid metabolism. *Parabacteroides distasonis*, a potential probiotic capable of converting primary to secondary bile acids, has shown therapeutic promise in several metabolic and inflammatory diseases. This study evaluated the preventive and therapeutic effects of *P. distasonis* against CDI and explored the underlying mechanisms. We characterized the probiotic properties of four *P. distasonis* strains and investigated the inhibitory activity of strain 1190003 against *C. difficile*, as well as its protective and therapeutic efficacy in mouse models. Gut microbiota structure and bile acid metabolic profiles were analyzed by integrating 16S rRNA gene sequencing and metabolomics. The four *P. distasonis* strains exhibited strong acid and bile salt tolerance as well as auto-aggregation ability. Supernatants from *P. distasonis* co-cultured with cholic acid (4 mM and 8 mM) significantly inhibited *C. difficile* growth, toxin expression and spore formation, with deoxycholic acid identified as the key inhibitory metabolite. Both live *P. distasonis* and its culture supernatant alleviated disease severity in CDI mouse models and ameliorated gut microbiota dysbiosis. Notably, the relative abundance of *Parabacteroides goldsteinii* was increased following supernatant treatment. Furthermore, intervention with either live *P. distasonis* or its supernatant elevated the level of hyodeoxycholic acid. In summary, *P. distasonis* acts as a potential probiotic that alleviates CDI by ameliorating gut microbiota dysbiosis and remodeling bile acid metabolism. These findings provide experimental evidence for its use as a microbiota-based therapeutic strategy.

## Graphical abstract

**Figure:**
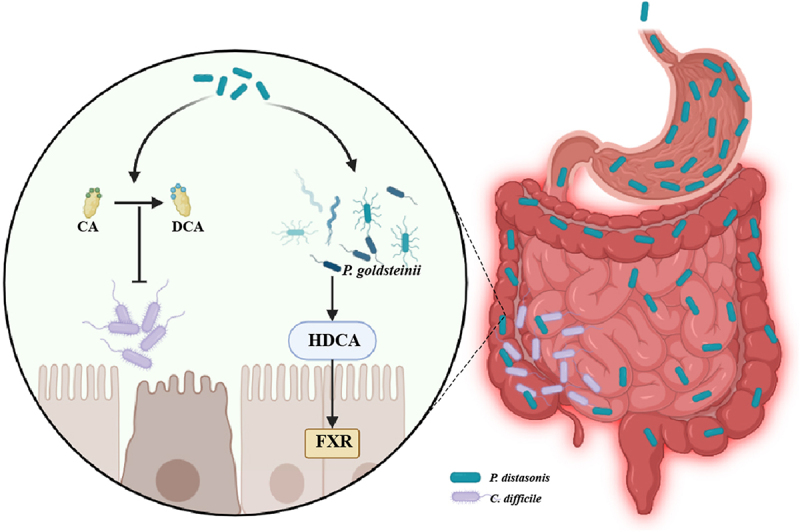

## Introduction

*Clostridioides difficile* (*C. difficile*) is a Gram-positive, obligate anaerobic, spore-forming bacillus. It is a leading cause of antibiotic-associated diarrhea and a major contributor to nosocomial infections. Broad-spectrum antibiotics disrupt gut microbial homeostasis, predisposing patients to *C. difficile* infection (CDI). CDI manifestations range from mild diarrhea to severe pseudomembranous colitis, toxic megacolon, bowel perforation, or even death [1,2]. Since the early 21st century, global CDI incidence has risen steadily. In 2017, the U.S. Centers for Disease Control and Prevention estimated 223,900 antibiotic-associated CDI cases and approximately 12,800 deaths in the United States [3,4].

Vancomycin, metronidazole, and fidaxomicin are first-line antibiotics for CDI treatment, but antibiotic therapy further disrupts the gut microbiota, leading to recurrence rates as high as 30% [5]. Vancomycin therapy may also cause adverse effects such as ototoxicity and nephrotoxicity [6]. Fecal microbiota transplantation (FMT), an effective treatment for recurrent CDI, inhibits *C. difficile* by restoring the gut microbiota and modulating microbial metabolites such as secondary bile acids [7]. CDI patients exhibit elevated levels of primary bile acids (e.g. cholic acid (CA) and chenodeoxycholic acid (CDCA)) and markedly reduced levels of secondary bile acids (e.g. deoxycholic acid (DCA) and lithocholic acid (LCA)) in the intestine. Secondary bile acids have been shown to inhibit *C. difficile* growth, spore germination, and toxin activity [8–11].

The antimicrobial properties of secondary bile acids have prompted the hypothesis that enrichment of secondary-bile-acid-producing bacteria could suppress *C. difficile*. Previous studies have shown that *Clostridium scindens* and *Clostridium hiranonis* produce secondary bile acids via the bile acid-inducible (*bai*) operon, thereby inhibiting *C. difficile* growth [12]. Notably, recent studies indicate that *Parabacteroides distasonis* (*P. distasonis*), although lacking a complete *bai* operon, participates in bile acid metabolism via bile salt hydrolases and steroid-transforming enzymes [13]. Beyond bile acid modulation, *P. distasonis* exerts immunomodulatory effects and has been reported to confer protection against a range of metabolic and inflammatory disorders, including obesity, colitis, rheumatoid arthritis, and hepatic fibrosis [14–16].

In this study, we evaluated the preventive and therapeutic potential of *P. distasonis* against CDI. We examined the inhibitory effects of *P. distasonis* on *C. difficile* growth, virulence gene expression, and spore formation in vitro, and assessed its efficacy in CDI mouse models. To elucidate underlying mechanisms, we integrated 16S rRNA gene sequencing and metabolomic profiling to characterize shifts in gut microbial composition and bile acid metabolism. This work provides a theoretical and experimental foundation for the development of *P. distasonis*-based microbiota therapies for CDI.

## Methods

### Bacterial strains, culture, and identification

Four *C. difficile* strains (ATCC BAA-1382, ATCC BAA-1870, ATCC 43255, and ATCC 43598) were obtained from the American Type Culture Collection (ATCC; Manassas, VA, USA). *C. difficile* was cultured anaerobically on selective *Clostridium difficile* moxalactam norfloxacin agar (CDMN; Oxoid, Cambridge, UK). Four *P. distasonis* strains (ATCC 8503, CGMCC 1.30169, 1190003, and F3-A 6JT) were used in this study. ATCC 8503 was obtained from ATCC; CGMCC 1.30169 was kindly provided by Prof. Shuangjiang Liu (Institute of Microbiology, Chinese Academy of Sciences); and strains 1190003 and F3-A 6JT were provided by Prof. Ruifu Yang (Academy of Military Medical Sciences, China). *P. distasonis* strains were cultured on enriched Brucella agar (BD Diagnostics, Franklin Lakes, USA) supplemented with 5 mg/L hemin and 1 mg/L vitamin K_1_. Colony identification was performed using matrix-assisted laser desorption ionization-time of flight mass spectrometry (MALDI-TOF MS; QuanTOF, IntelliBio, Qingdao, China), as described previously [17,18]. All bacterial strains were preserved in 15% (v/v) glycerol at −80°C until use (strain information listed in Table S1).

### Bile acid tolerance and antibiotic susceptibility testing

Bile acid tolerance and antibiotic susceptibility were determined following the guidelines of the Clinical and Laboratory Standards Institute (CLSI; Malvern, PA, USA; M11-A7). Agar dilution assays were used to determine the tolerance of *P. distasonis* strains to CA, DCA and hyodeoxycholic acid (HDCA), and to assess their susceptibility to ceftriaxone, ceftazidime, vancomycin, meropenem, tetracycline, erythromycin, fidaxomicin, levofloxacin, ciprofloxacin, clindamycin, linezolid, rifampin, and penicillin. Minimum inhibitory concentrations (MICs) were interpreted according to CLSI standards.

### Acid and taurocholate tolerance assays

Acid and taurocholate tolerance was assessed as previously described with minor modifications [19]. Briefly, overnight cultures of the four *P. distasonis* strains were inoculated (1:100) into fresh BHIS broth (brain heart infusion supplemented with 5% yeast extract and 0.1% cysteine). Optical density at 620 nm (OD_620_) was monitored using a Multiskan FC microplate reader (Thermo Fisher Scientific, Waltham, MA, USA) until mid-log phase (OD_620_ = 0.5). Cultures were then transferred into BHIS broth adjusted to pH 3–6 or supplemented with 0.1–0.5% (w/v) taurocholate, and incubated anaerobically at 37°C for 3 h. Survival rate was calculated as the ratio of colony-forming units (CFUs) after 3 h to CFUs at 0 h.

### Auto-aggregation assay

Auto-aggregation was evaluated following Patel et al. [20] with modifications. Overnight cultures were centrifuged at 4,800 × g for 10 min, washed twice with sterile phosphate buffered saline (PBS), and resuspended. The absorbance was measured at 620 nm and recorded as *A*_0_. 5 mL of the suspension was incubated statically at 37°C in sterile glass tubes. Supernatant absorbance (*A*_t_) was measured at 6, 12, and 24 h. The auto-aggregation rate was calculated as [(*A*_0_−*A*_t_)/*A*_0_] × 100%.

### Preparation of cell-free supernatants and untargeted metabolomic analysis

Cell-free supernatants were prepared as described by Reed et al. [12] with minor modifications. *P. distasonis* was cultured in BHIS broth supplemented with 0 mM, 0.5 mM, 1 mM, 2 mM, 4 mM, or 8 mM CA under anaerobic conditions for 24 h. Cultures were centrifuged (4,500 × g, 20 min, 4°C), and supernatants were filtered through 0.22 µm filters. The resulting sterile supernatant was mixed with fresh BHIS broth at a 2:1 ratio for subsequent inhibitory assays (hereinafter referred to as mixed broth).

Untargeted metabolomic profiling was performed by Personalbio (Shanghai, China) using a Vanquish Flex UHPLC system coupled with an Orbitrap Exploris 120 mass spectrometer (Thermo Fisher Scientific, Waltham, MA, USA). Chromatographic separation employed an ACQUITY UPLC HSS T3 column with a linear gradient of mobile phase A (0.1% formic acid in water) and mobile phase B (acetonitrile with 0.1% formic acid). Data were acquired in positive and negative ion modes using heated electrospray ionization (HESI), with spray voltages of 3.5 kV and −3.0 kV, a resolution of 60,000 (MS1), and an m/z range of 70–1000. Data processing, including peak alignment and metabolite identification, was performed using MS-DIAL software.

### Growth kinetics assay

Growth kinetics were measured as described by Reed et al. [12] with modifications. *C. difficile* overnight cultures were inoculated (1:100) into fresh BHIS broth and grown to mid-log phase (OD_620_ = 0.5). Cultures were then transferred into mixed broth and incubated anaerobically. OD_620_ was measured every 2 h for 16 h, with 10 s of shaking before each reading. Log-phase samples were collected for subsequent RNA extraction.

### Quantification of spore formation

Spore quantification followed previously published protocols with minor modifications [21]. Briefly, *C. difficile* cultures were inoculated (1:100) into fresh BHIS broth and grown to mid-log phase. 100 μL of the culture was transferred into mixed broth and incubated anaerobically for 5 days. Daily samples (1 mL) were collected, centrifuged at 3,500 rpm for 10 min, washed with sterile PBS, heated at 65°C for 30 min, and plated on BHIS agar containing 0.1% sodium taurocholate. CFUs were counted after 48 h of incubation at 37°C.

### Biofilm formation assay

Biofilm formation was assessed as previously described [21] with modifications. *C. difficile* cultures (1:100) were inoculated into mixed broth containing 1% glucose, transferred to 96-well plates, and incubated anaerobically at 37°C for 72 h and 120 h. After incubation, wells were washed twice with sterile PBS, air-dried, and stained with 0.2% (w/v) crystal violet for 30 min. Excess dye was removed, and wells were rinsed twice with sterile PBS. After drying, 200 µL/well of 95% ethanol was added for destaining, and the optical density was measured at 572 nm. BHISG (BHIS supplemented with 1% glucose) medium without bacteria served as a blank control.

### RNA extraction and quantitative real-time PCR

We performed gene expression analysis according to the method previously described [22]. Total RNA was extracted from log-phase *C. difficile* cultures using a bacterial RNA extraction kit (Vazyme, Nanjing, China). Contaminating genomic DNA was removed and cDNA was reverse transcribed using the HisyGo RT Red SuperMix kit (Vazyme). Quantitative real-time PCR (RT-qPCR) was performed using ChamQ Universal SYBR qPCR Master Mix (Vazyme). Expression levels were normalized to the reference gene 16S rRNA. The expression levels of *tcdA*, *tcdB*, and *spo0A* were quantified, and relative expression was calculated using the 2^–ΔΔCt^ method [23]. Primer sequences are listed in Table S2.

### Co-incubation of cholestyramine with supernatant

The interaction between cholestyramine resin and *P. distasonis* supernatant was assessed as described by Mangnall et al. [24] with modifications. Cholestyramine resin (ion exchange capacity: 1.0 meq/g) was added in 5–10-fold molar excess to ensure complete binding. The supernatant and resin mixture was incubated at 37°C for 1 h with agitation, followed by centrifugation at 3,000 × g for 25 min, and supernatants were filtered through 0.22 µm filters.

### Mice

Six-week-old SPF-grade male C57BL/6N mice (Weitong Lihua, Beijing, China) were housed under controlled conditions (22–25°C, 12 h light/dark cycle) with free access to standard chow and ultrapure water. All animal experiments in this study were performed according to the Guide for the Care and Use of Laboratory Animals (Institute of Laboratory Animal Resources, National Research Council, United States), with ethical approval obtained from the Ethics Committee of the Second Hospital of Hebei Medical University (approval number: 2024-AE247). All experimental procedures were performed in accordance with the ARRIVE Guidelines 2.0.

### CDI mouse model

The CDI mouse model was established as described by Chen et al. [25] with modifications. Mice were pretreated with a cocktail of antibiotics (0.215 mg/mL metronidazole, 0.4 mg/mL kanamycin, 0.035 mg/mL gentamicin, 0.05675 mg/mL polymyxin B, and 0.045 mg/mL vancomycin) administered in drinking water for 5 days, followed by regular water. 24 h prior to infection, clindamycin (100 mg/kg) was administered intraperitoneally. On day 0, all mice except those in the normal control (NC) group were orally gavaged with *C. difficile* ATCC BAA-1870 (10^8^ CFU). For therapeutic intervention, the live *P. distasonis* (PD) group received *P. distasonis* 1190003 (10^8^ CFU) via oral gavage. The *P. distasonis* cell-free supernatant (PD CFS) group received the cell-free supernatant prepared as follows. *P. distasonis* was co-cultured with 4 mM CA for 24 h, followed by centrifugation and filtration as described above to obtain the supernatant, and each mouse received 200 μL via oral gavage. Both PD and PD CFS interventions were administered prophylactically, starting from the first day of antibiotic treatment and continuing until the end of the experiment. The vancomycin (VAN) group received vancomycin (50 mg/kg) via oral gavage starting from day 1 post-infection. Body weight and disease symptoms were recorded daily. On day 7, mice were euthanized by CO_2_ asphyxiation, and tissues were collected.

Two independent mouse experiments were conducted to evaluate the therapeutic effect of *P. distasonis* against CDI. For Experiment 1, mice were randomly assigned to four groups based on body weight: NC group (*n* = 5), CDI group (*n* = 5), VAN group (*n* = 5), and PD group (*n* = 7). For Experiment 2, mice were randomly assigned to four groups based on body weight (*n* = 8 per group): NC, CDI, PD, and PD CFS group.

### Quantification of cecal spore load

Cecal spore counts were determined following Buffie et al. [26] with minor modifications. Approximately 20 mg of cecal contents was treated with absolute ethanol for 1 h to eliminate vegetative cells. After centrifugation, the pellet was resuspended in sterile PBS, heated at 65°C for 30 min, and plated on CDMN agar containing 0.1% sodium taurocholate. Plates were incubated anaerobically at 37°C for 48 h before colony counting.

### Relative body weight and clinical sickness scoring

Body weight was recorded daily starting from the infection day (day 0). Relative body weight (RBW) was calculated as [(*W*_t_ / *W*_0_) × 100%] averaged across all surviving mice in the group, where *W*_t_ is the body weight of an individual mouse on day t, and *W*_0_ is the body weight of the same mouse on day 0 prior to *C. difficile* challenge. Clinical sickness scores (CSS) were assigned based on stool consistency, behavior, and body weight loss. Each category was scored from 0 to 4 (Table 1), as previously described [27].

**Table 1.** Clinical sickness scoring (CSS) [27].

Modified bristol stool scoring		Behavior		Weight loss	
0	Sausage shaped, lumpy or with cracks on surface	0	Able to ambulate, normal posture, eyes open, explores cage freely	0	≥100% original weight
1	Sausage/snake-like, smooth and soft texture	1	Sluggish ambulation & hunched posture, still moving around cage	1	96–99% original weight
2	Soft blobs or fluffy pieces, easily passable	2	Sluggish ambulation, hunched posture, little spontaneous ambulation	2	91–95% original weight
3	Entirely liquid stool	3	Only ambulates with stimulation, hunched posture, eyes closed	3	86–90% original weight
4	Mucous stool	4	Unable to ambulate, hunched posture, eyes closed, unresponsive or dead	4	≤85% original weight

Note: Combined final scores for each mouse range from 0 to 12, with the total score recorded in the final analysis.

### Histopathological analysis (H&E and PAS staining)

Colonic tissues were fixed in 4% paraformaldehyde for 24 h, transferred to 75% ethanol, and embedded in paraffin. Sections (4 μm) were stained with hematoxylin and eosin (H&E) and periodic acid-Schiff (PAS) for histological evaluation. In a blinded manner, two pathologists independently scored the sections based on neutrophil infiltration, hemorrhage/congestion, mucosal edema, and epithelial damage (0–3 each; total 0–12) [28]. Goblet cell abundance was evaluated in PAS-stained sections. Images were captured under an Olympus BX61 microscope (×100), and analyzed with Fiji (ImageJ).

### Immunohistochemistry (IHC)

Paraffin-embedded colon sections underwent deparaffinization, rehydration, and heat-induced antigen retrieval. Endogenous peroxidase was blocked with 3% H_2_O_2_ for 15 min, followed by 10% goat serum for 1 h. Sections were incubated overnight at 4°C with primary antibodies against Muc2 (1:500) and ZO-1 (1:100) (Servicebio, Wuhan, China), then with HRP-conjugated secondary antibody, and visualized using DAB (Servicebio). Nuclei were counterstained with hematoxylin. Images were captured and analyzed as described above.

### Tissue RNA extraction and qPCR analysis

Approximately 20 mg of colon tissue was homogenized on ice, and total RNA was extracted using a tissue RNA extraction kit (Vazyme). Reverse transcription and qPCR were performed as described above. Expression levels were normalized to the reference gene GAPDH. Primer sequences are listed in Table S3.

### 16S rRNA gene sequencing and microbiota analysis

Cecal contents were collected for 16S rRNA gene sequencing (V3-V4 region) performed by Personalbio. DNA was extracted using the MagBeads FastDNA Kit for Soil (MP Biomedicals, CA, USA). PCR amplification was conducted using primers 338F (5’-ACTCCTACGGGAGGCAGCA-3’) and 806R (5’-GGACTACHVGGGTWTCTAAT-3’). Libraries were sequenced on an Illumina NovaSeq 6000 platform. Raw data were processed with QIIME2 using demux, cutadapt, and DADA2 plugins for demultiplexing, trimming, denoising, and chimera removal. Amplicon sequence variants (ASVs) were clustered at 100% similarity, and abundance tables were generated.

### Cecal bile acid quantification

Approximately 100 mg of cecal contents per mouse was analyzed for bile acid composition by Personalbio. After sample preparation, bile acids were quantified using LC-MS/MS (ExionLC^TM^ AD system coupled with QTRAP 6500+ mass spectrometer; Sciex, Framingham, MA, USA). Calibration curves for individual bile acids were constructed, and quantitative data were processed using MultiQuant 3.0.3 software.

### Statistical analysis

All experiments were performed in triplicate with three biological replicates. Data are presented as mean ± standard deviation (SD). Biofilm formation, auto-aggregation, gene expression, colony counts, toxin quantification, and histopathology scores were analyzed by one-way analysis of variance (ANOVA). Spore quantification, body weight, and clinical sickness scores were assessed by two-way ANOVA (IBM SPSS Statistics 21.0). Graphs were generated using GraphPad Prism 9.5.1. Differences were considered statistically significant at *p* < 0.05.

## Results

### *P. distasonis* strains exhibit tolerance to acid, bile salts, and antibiotics

Colonization and survival in the human gastrointestinal tract are fundamental prerequisites for probiotics. This study evaluated the tolerance of four *P. distasonis* strains to varying pH levels and bile salt concentrations, as well as their auto-aggregation capacity.

**Figure 1.**
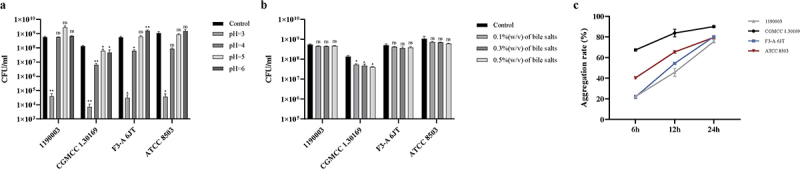
Acid and bile salt tolerance and auto-aggregation of the four *P. distasonis* strains. (a) Viability after 3 h incubation under acidic conditions (pH 3.0–6.0). (b) Viability after 3 h exposure to varying concentrations of bile salts (0.1–0.5%). (c) Kinetics of auto-aggregation. Data are presented as mean ± SD, analyzed by one-way ANOVA and Bonferroni post-hoc test. *, *p* < 0.05; **, *p* < 0.01; ns, not significant.

All four strains exhibited strong adaptability to environmental stresses after exposure to pH 3–6 and 0.1–0.5% bile salts. At pH 3, bacterial viability was markedly reduced; however, at pH ≥4, survival rates increased significantly, with strain 1190003 showing the highest tolerance. After 3 h of exposure to 0.1–0.5% bile salts, the viability of all strains remained largely unaffected (Figure 1(a,b)). Moreover, all strains exhibited substantial auto-aggregation ability, with strain CGMCC 1.30169 demonstrating the strongest capability, achieving an auto-aggregation rate of 67.36% after 6 h (Figure 1(c)).

According to CLSI guidelines, the antibiotic susceptibility of the four *P. distasonis* strains was evaluated against 12 antimicrobial agents. All strains exhibited resistance to most antibiotics commonly used against Gram-positive bacteria, including the frontline CDI therapeutics metronidazole, vancomycin, and fidaxomicin (Table 2).

**Table 2.** Mics of antimicrobial agents for *P. distasonis* strains.

Antibiotics	MICs (µg/ml)			
	1190003	CGMCC 1.30169	F3-A 6JT	ATCC 8503
Cefepime	>8	0.5	>8	2
Meropenem	0.5	<0.064	4	<0.064
Vancomycin	>4	1	>4	>4
Metronidazole	2	1	2	1
Fidaxomicin	>8	>8	>8	>8
Erythromycin	>2	>2	>2	1
Tetracycline	<0.5	<0.5	16	<0.5
Levofloxacin	>16	<0.5	0.125	2
Clindamycin	>2	>2	>2	<0.064
Linezolid	1	1	1	2
Rifampin	<0.25	<0.25	<0.25	<0.25
Penicillin	>16	16	>16	0.5

### Supernatants from *P. distasonis* cultured with CA effectively suppress the pathogenicity of *C. difficile*

The pathogenic phenotypes of four *C. difficile* strains (ATCC BAA-1382, ATCC BAA-1870, ATCC 43255, and ATCC 43598) were evaluated after exposure to supernatants from *P. distasonis* cultures supplemented with varying concentrations of CA. The assessed parameters included bacterial growth kinetics, biofilm formation, auto-aggregation, toxin production, and spore formation.

**Figure 2.**
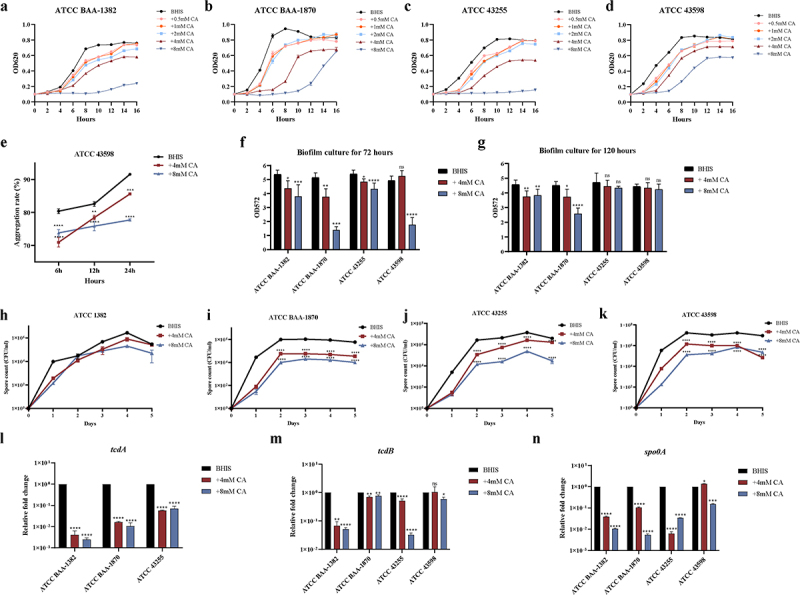
Inhibitory effects of supernatants from *P. distasonis* co-cultured with 4 mM or 8 mM CA on pathogenic phenotypes of *C. difficile*. Growth curves of *C. difficile* strains ATCC BAA-1382 (a), ATCC BAA-1870 (b), ATCC 43255 (c), and ATCC 43598 (d). (e) Auto-aggregation rates of *C. difficile* ATCC 43598. Biofilm formation at 72 h (f) and 120 h (g). Spore counts of *C. difficile* ATCC BAA-1382 (h), ATCC BAA-1870 (i), ATCC 43255 (j), and ATCC 43598 (k). Relative mRNA expression levels of *tcdA* (l), *tcdB* (m) and *spo0A* (n) in *C. difficile*. Data are presented as mean ± SD, analyzed by one-way ANOVA and Bonferroni post-hoc test. Two-way ANOVA was used for auto-aggregation and spore count data. *, *p* < 0.05; ***, *p* < 0.001; ****, *p* < 0.0001; ns, not significant.

All four *P. distasonis* strains exhibited strong tolerance to CA (Table S4). Among the four *P. distasonis* strains tested in Figure S1 and Figure S2, strain 1190003 exhibited the strongest inhibitory effect against *C. difficile* and was therefore selected for subsequent experiments. Notably, neither CA alone nor the supernatant of *P. distasonis* grown without CA significantly suppressed *C. difficile* growth (Figure S1). When co-cultured with CA, supernatants from 4 mM and 8 mM treatments markedly inhibited *C. difficile* growth, whereas those from 0.5 mM, 1 mM, and 2 mM CA treatments showed no significant effect compared to the control (Figure 2(a–d)). Consequently, supernatants from *P. distasonis* co-cultured with 4 mM or 8 mM CA were used in subsequent assays.

To investigate the effect of supernatants on *C. difficile* adhesion and colonization, we assessed their impact on auto-aggregation and biofilm formation. For auto-aggregation, all four tested strains showed significant inhibition of auto-aggregation when treated with supernatants, with the 8 mM CA-conditioned supernatant exhibiting the strongest effect (Figure 2(e), S3(a-c)). Among the four strains, *C. difficile* ATCC 43598 displayed the highest auto-aggregation ability, which is consistent with a previous report [21]. Therefore, this strain was selected for subsequent auto-aggregation assays. For biofilm formation, we examined the effect of supernatants at 72 h and 120 h (Figure 2(f,g)). At 72 h, 8 mM CA-conditioned supernatants significantly reduced biofilm formation. At 120 h, the inhibition became less evident. The expression levels of biofilm-related genes (*pilA1*, *pilB1*, *slpA*, *fliC*, *fliD*, and *cwp84*) were examined (Figure S3d). Although an upward trend was observed in gene expression following supernatant treatment, the differences were not statistically significant.

The impact of the supernatants on spore formation was further evaluated over a five-day monitoring period (Figure 2(h–k)). Both 4 mM and 8 mM CA-conditioned supernatants significantly suppressed spore formation in strains ATCC BAA-1870, ATCC 43255, and ATCC 43598. However, for strain ATCC BAA-1382, which inherently exhibits low sporulation capacity, the treatment had no significant effect.

Finally, transcriptional analysis revealed that the CA-conditioned supernatants markedly downregulated the expression of toxin genes (*tcdA* and *tcdB*) and the sporulation master regulator *spo0A* (Figure 2(l–n)).

Collectively, these results demonstrate that supernatants from *P. distasonis* co-cultured with CA significantly inhibit *C. difficile* growth and virulence-associated phenotypes, with stronger effects observed in the 8 mM CA group.

### The antibacterial activity of the supernatant depends on bile acid metabolites

**Figure 3.**
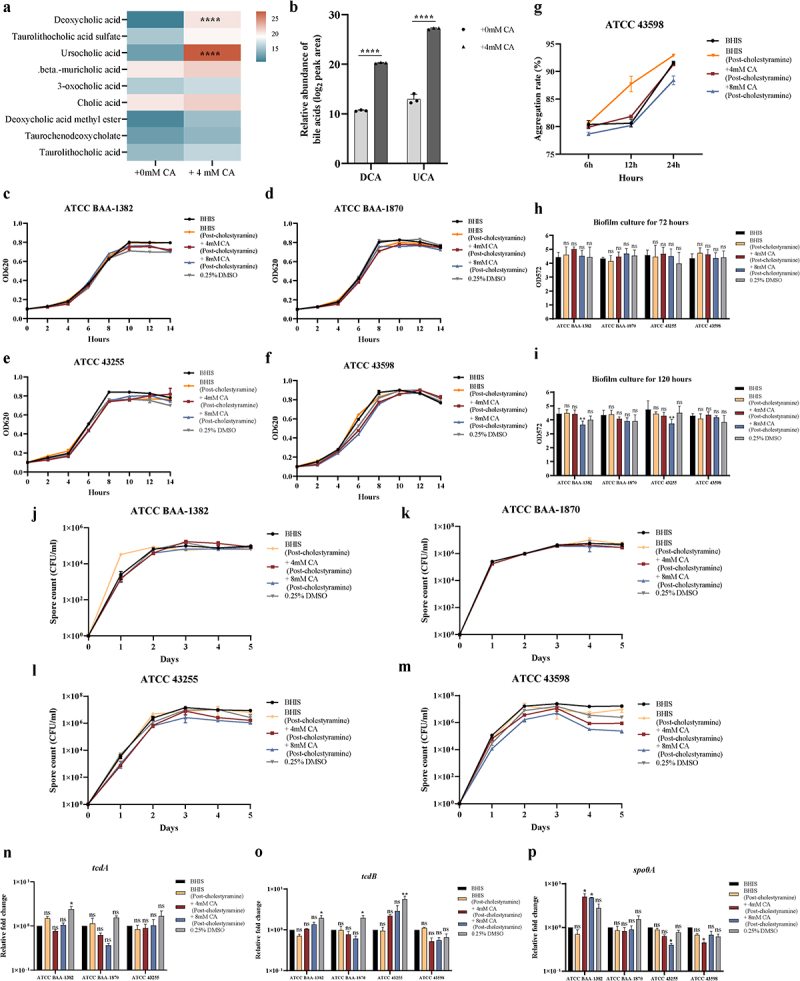
Cholestyramine treatment reduces the inhibitory effects of supernatants from *P. distasonis* co-cultured with CA on *C. difficile*. (a, b) Relative abundance of bile acids in the supernatant of *P. distasonis* before and after CA supplementation. Growth curves of *C. difficile* strains ATCC BAA-1382 (c), ATCC BAA-1870 (d), ATCC 43255 (e), and ATCC 43598 (f). (g) Auto-aggregation rates of *C. difficile* ATCC 43598. Biofilm formation at 72 h (h) and 120 h (i). Spore counts of *C. difficile* ATCC BAA-1382 (j), ATCC BAA-1870 (k), ATCC 43255 (l) and ATCC 43598 (m). Relative mRNA expression levels of *tcdA* (n), *tcdB* (o) and *spo0A* (p) in *C. difficile*. Data are presented as mean ± SD, analyzed by one-way ANOVA and Bonferroni post-hoc test. Two-way ANOVA was used for auto-aggregation and spore count data. *p < 0.05; **p < 0.01; ***p < 0.001; ns, not significant.

To identify the key inhibitory components in the *P. distasonis* supernatant, we analyzed the compositional changes before and after CA supplementation. Following co-culture with 4 mM CA, secondary bile acid levels increased markedly (Figure 3(a,b)). Notably, DCA and ursocholic acid (UCA) were significantly elevated, confirming that *P. distasonis* possesses the capacity to convert primary bile acids to secondary bile acids in vitro.

We next used cholestyramine, a bile acid sequestrant, to remove bile acids from the supernatant and evaluated its inhibitory effects on virulence factors of *C. difficile*. Cholestyramine is practically insoluble in water but soluble in dimethyl sulfoxide (DMSO), a solvent control group (DMSO alone) was included. As expected, cholestyramine treatment markedly attenuated or abolished the supernatant’s bacteriostatic and antivirulence effects. The treated supernatants no longer inhibited *C. difficile* growth (Figure 3(c–f)), auto-aggregation (Figure 3(g)), biofilm formation (Figure 3(h,i)), or spore formation (Figure 3(j-m)). The inhibitory effects were further confirmed at the transcriptional level (Figure 3(n-p)).

### Deoxycholic acid directly suppresses C. difficile pathogenicity

**Figure 4.**
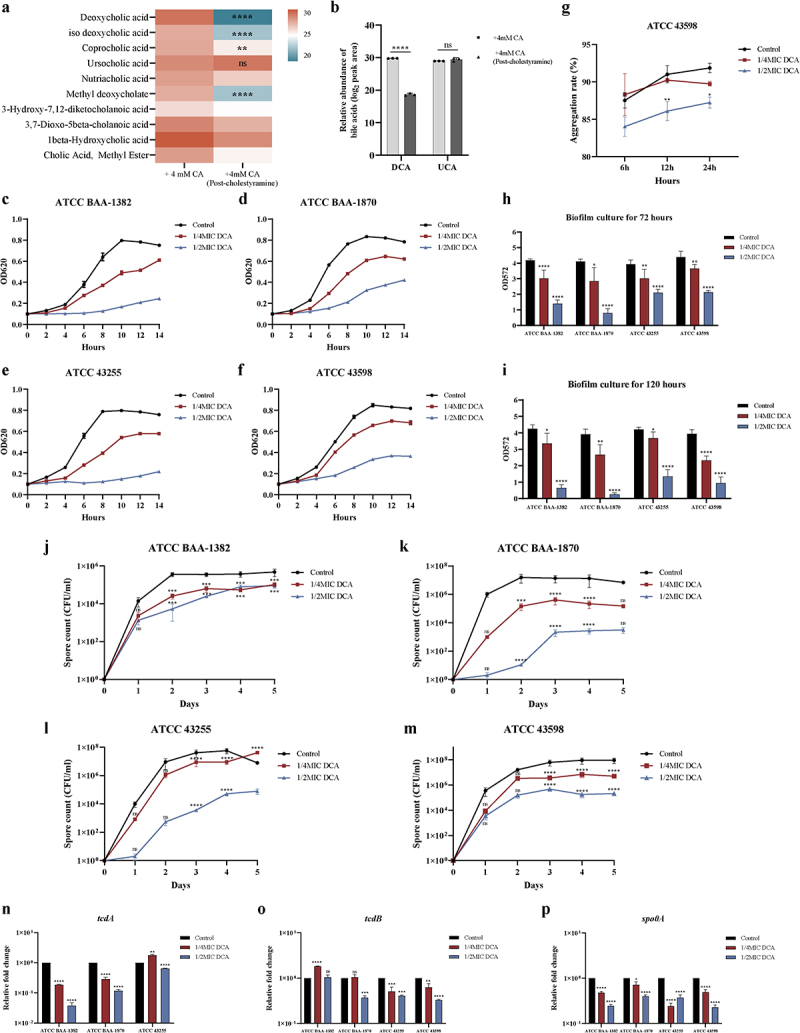
Deoxycholic acid is the primary antimicrobial component in the supernatant that inhibits *C. difficile*. (a, b) Relative abundance of bile acids in the supernatant of *P. distasonis* before and after cholestyramine treatment. (c–p) Effects of DCA at 1/2 MIC and 1/4 MIC on growth curves, auto-aggregation, biofilm formation, and spore formation of four *C. difficile* strains. Data are presented as mean ± SD, analyzed by one-way ANOVA and Bonferroni post-hoc test. Two-way ANOVA was used for auto-aggregation and spore count data. *p < 0.05; **p < 0.01; ***p < 0.001; ns, not significant.

To identify the bile acid species primarily responsible for the inhibitory activity, we analyzed the supernatant composition before and after cholestyramine treatment. Cholestyramine treatment markedly altered the bile acid profile and abundance (Figure 4(a,b)). The level of DCA was significantly reduced, whereas UCA levels remained unchanged, suggesting that DCA is the principal bile acid component mediating the inhibitory effects on *C. difficile*.

All four tested *C. difficile* strains exhibited a MIC of 1 mM for DCA (Table S4). Subsequent treatments were performed with sub-MIC concentrations (1/2 MIC and 1/4 MIC). As shown in Figure 4(c-p), DCA significantly inhibited growth, auto-aggregation, biofilm formation, and spore formation of all strains in a concentration-dependent manner.

### *Live P*. distasonis administration alleviates CDI in mice

**Figure 5.**
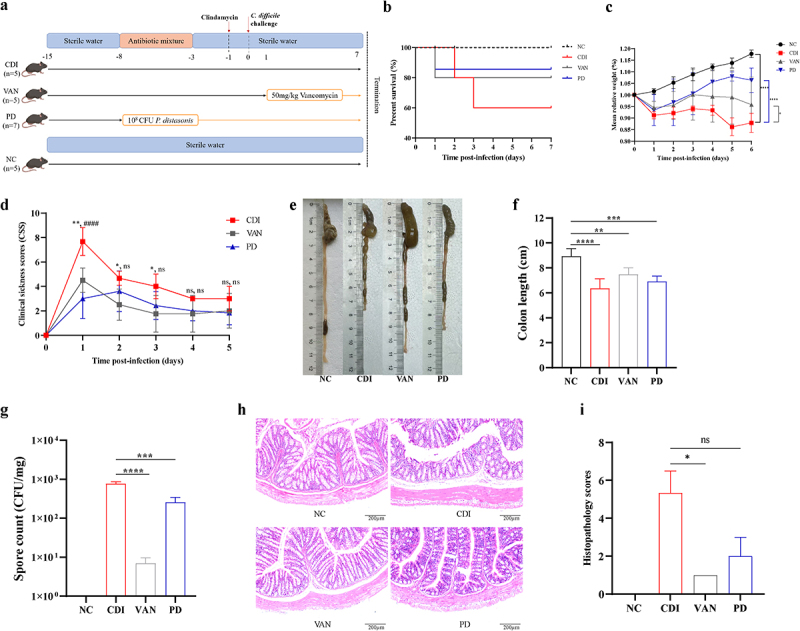
Live *P. distasonis* ameliorates CDI in mice. Normal control group (NC, *n* = 5), *C. difficile* infection group (CDI, *n* = 5), vancomycin treatment group (VAN, *n* = 5), live *P. distasonis* intervention group (PD, *n* = 7). (a) Experimental scheme. Mice were pretreated with a cocktail of antibiotics administered in drinking water for 5 days. 24 h prior to infection, clindamycin (100 mg/kg) was administered intraperitoneally. On day 0, mice in the infection group were orally gavaged with *C. difficile* ATCC BAA-1870 (10^8^ CFU). (b) Percent survival. (c) Mean relative weight. Statistical comparisons were performed on day 5. (d) Clinical sickness score. *, CDI vs VAN; #, CDI vs PD. (e) Representative cecal morphology. (f) Colon length measurement. (g) *C. difficile* spore load in cecal contents. (h) Representative H&E pathological staining images of colon sections on day 7 (100×). Scale bar: 200 μm. (i) Histopathology scores (*n* = 3). Data are presented as mean ± SD, analyzed by one-way ANOVA and Bonferroni post-hoc test. Two-way ANOVA was used for mean relative weight and clinical sickness scores data. Survival curves were analyzed by the log-rank test. *p < 0.05; **p < 0.01; ***p < 0.001; ****p < 0.0001; ####*p* < 0.0001; ns, not significant.

We investigated the protective effect of PD in CDI mice, as illustrated in Figure 5(a). Compared with the CDI group, administration of PD improved the survival rate of infected mice (85.7% vs 60%), although the difference was not statistically significant (*p* = 0.865) (Figure 5(b)). PD also mitigated body weight loss (Figure 5(c)) and alleviated disease symptoms (Figure 5(d)).

Mice in the CDI group exhibited marked cecal hyperemia, enlargement, and colonic shortening. These parameters were improved in the PD-treated group, but none reached statistical significance compared with the CDI group (Figures 5(e,f)). The murine cecum is large with thoroughly mixed contents, allowing quantitative analysis with minimal inter-individual variation. Therefore, we quantified *C. difficile* spore counts in cecal contents. The results further demonstrated that both vancomycin and PD significantly reduced *C. difficile* spore burden (Figure 5(g)). Histopathological analysis of colonic tissues (H&E staining) showed that CDI mice exhibited mucosal atrophy, expansion of the submucosal layer, and marked inflammatory cell infiltration. In contrast, PD-treated mice maintained epithelial integrity, showed reduced inflammation, and had lower histopathology scores (Figure 5(h,i)). Collectively, PD effectively alleviates the severity of CDI and protects intestinal tissue integrity.

### *P. distasonis* cell-free supernatant alleviates CDI in mice

**Figure 6.**
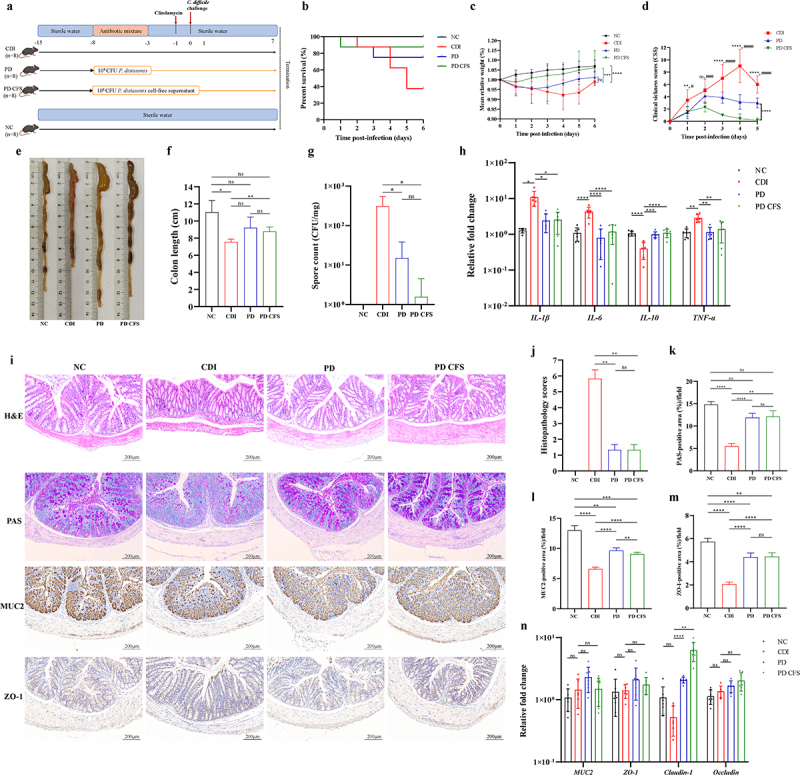
*P. distasonis* cell-free supernatant ameliorates CDI in mice. Normal control group (NC), *C. difficile* infection group (CDI), live *P. distasonis* intervention group (PD), *P. distasonis* cell-free supernatant intervention group (PD CFS), *n* = 8. (a) Experimental scheme. Mice in the PD group were orally gavaged with live *P. distasonis* 1190003 (10^8^ CFU), while mice in the PD CFS group were orally administered the supernatant collected after 24 h co-culture of *P. distasonis* 1190003 with 4 mM CA. (b) Percent survival. (c) Mean relative weight. Statistical comparisons were performed on day 5. (d) Clinical sickness score. *, CDI vs PD; #, CDI vs PD CFS. (e) Representative cecal morphology. (f) Colon length measurement. (g) *C. difficile* spore load in cecal contents. (h) Inflammatory cytokine mRNA expression (*n* = 7). (i) Representative images of H&E, PAS, and immunohistochemical staining for MUC2, ZO-1 in colon sections on day 7 (100×). Scale bar: 200 μm. (j) Histopathology scores (*n* = 3). (k) PAS-positive area (%) per field (*n* = 3). (l, m) MUC2 and ZO-1 positive area (%) per field (*n* = 3). (n) Intestinal barrier protein mRNA expression (*n* = 7). Data are presented as mean ± SD, analyzed by one-way ANOVA and Bonferroni post-hoc test. Two-way ANOVA was used for mean relative weight and clinical sickness scores data. Survival curves were analyzed by the log-rank test. *p < 0.05; **p < 0.01; ***p < 0.001; ****p < 0.0001; ###*p* < 0.001; ####*p* < 0.0001; ns, not significant.

We next investigated the therapeutic efficacy of PD CFS in CDI mice, as illustrated in Figure 6(a). PD CFS treatment alleviated CDI symptoms, as demonstrated by improved survival (*p* = 0.1767) (Figure 6(b)), reduced weight loss with rapid recovery (Figure 6(c)), and consistently lower clinical sickness scores throughout the experiment (Figure 6(d)). Moreover, PD CFS treatment markedly reduced *C. difficile* spore loads in cecal contents (Figure 6(g)) and alleviated CDI-induced intestinal pathological damage, as evidenced by ameliorated cecal swelling and colonic shortening (Figures 6(e,f)).

Histological analysis was performed to evaluate the protective effects of PD CFS on intestinal integrity and mucosal barrier function. As shown in Figure 6(i), H&E staining revealed that PD CFS treatment reduced damage to the colonic mucosal epithelium and decreased inflammatory cell infiltration, with significantly improved histopathology scores (Figure 6(j)). PAS staining showed that goblet cells and mucin granules, which were significantly reduced in the CDI group, were restored following PD CFS treatment, with statistically significant differences compared with the CDI group (Figure 6(k)). Immunohistochemical staining further demonstrated that PD CFS intervention significantly upregulated the expression of colonic mucin-2 (MUC2) and zonula occludens-1 (ZO-1) (Figure 6(l,m)). Compared with the CDI group, the mRNA level of *Claudin-1* was significantly increased in both the PD and PD CFS groups; however, the mRNA levels of *Muc2*, *ZO-1*, and *Occludin* in the PD and PD CFS groups showed increasing trends, though the differences were not statistically significant (Figure 6(n)).

PD CFS treatment also modulated inflammatory responses. As shown in Figure 6(h), mRNA expression levels of proinflammatory cytokines, including IL-1β, IL-6, and TNF-α, were downregulated in both the PD and PD CFS groups, while the expression of the anti-inflammatory cytokine IL-10 was upregulated, indicating that PD CFS possesses immunomodulatory effects.

### Treatment with live *P. distasonis* and its supernatant restores gut microbiota dysbiosis and bile acid metabolic homeostasis

**Figure 7.**
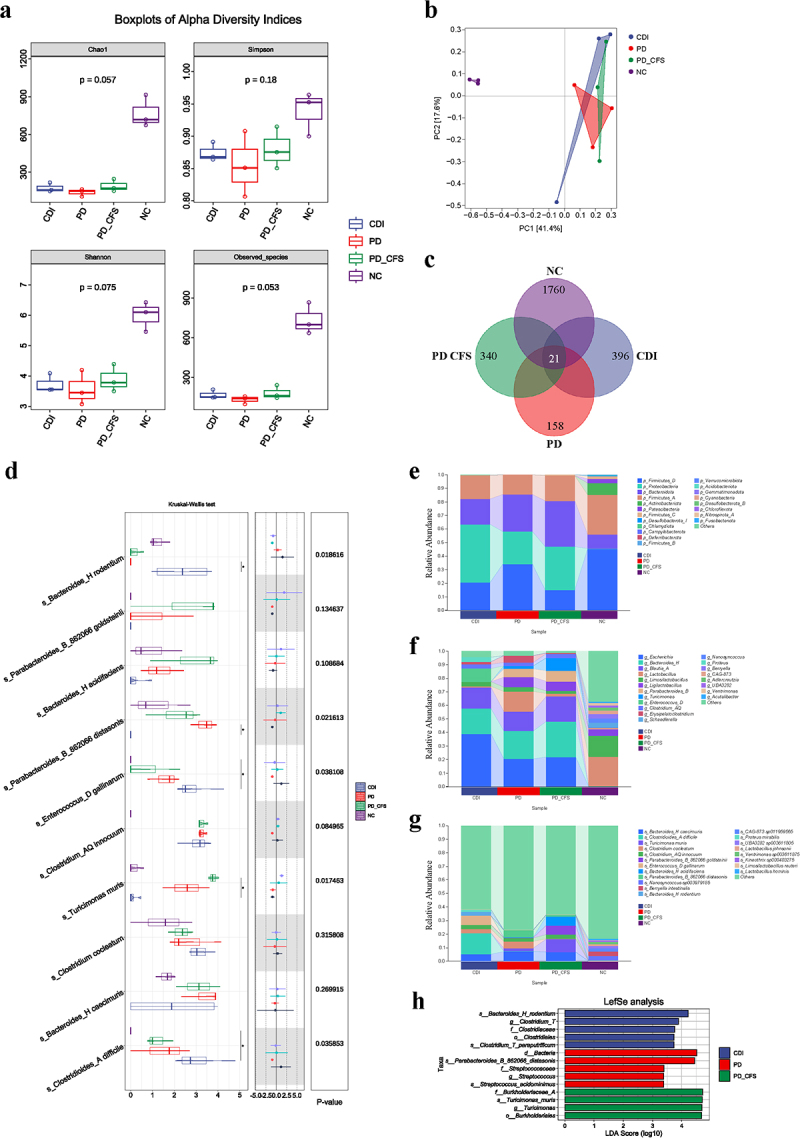
*P. distasonis* restores gut microbiota dysbiosis in CDI mice. Normal control group (NC), *C. difficile* infection group (CDI), live *P. distasonis* intervention group (PD), *P. distasonis* cell-free supernatant intervention group (PD CFS), *n* = 3 (a) α-diversity. (b) PCoA analysis of β-diversity. (c) Venn diagram. (d) Species difference analysis, the left column shows the converted abundance values; the middle column indicates the post hoc test method; and the right column displays the corresponding *p* values (*n* = 3). (e-g) Phylum, genus and species levels composition, the species composition was analyzed by presenting the top 20 most abundant taxa. (h) LEfSe analysis. The LEfSe bar plot displays taxa with significant differences among groups. The x-axis represents the LDA score (log10), and taxa are ranked by the magnitude of this score. LDA score threshold of 3 was set for discriminative features. Statistical significance was determined by Kruskal-Wallis test, post hoc analysis was performed using Dunn’s test. *p < 0.05, ns, not significant.

We performed 16S rRNA sequencing of mouse cecal contents, which revealed significant intergroup differences in microbial structure and diversity. In terms of α-diversity, the PD CFS group showed increased microbial richness, although the differences were not statistically significant (Figure 7(a)). Principal coordinate analysis (PCoA) based on Bray-Curtis distances revealed distinct separation between the CDI and NC groups, while the PD and PD CFS groups shifted microbial profiles away from the CDI group (Figure 7(b)). The Venn diagram revealed an increased number of unique operational taxonomic units (OTUs) in the CDI group (Figure 7(c)).

At the phylum level, the CDI group exhibited reduced *Firmicutes* and *Bacteroidetes*, accompanied by an increase in *Proteobacteria* abundance. Treatment with either PD or PD CFS partially alleviated this dysbiosis, with PD showing a slightly stronger effect on microbial reconstitution (Figure 7(e)). At the genus level, beneficial taxa such as *Bacteroides* and *Lactobacillus* were enriched, while potentially pathogenic genera such as *Escherichia* were reduced in both treatment groups (Figure 7(f)). At the species level, the relative abundance of *C. difficile* was significantly decreased in both PD and PD CFS groups (Figure 7(g)). Species composition difference and LEfSe analyses further demonstrated enrichment of *Parabacteroides goldsteinii* in the PD CFS group, whereas opportunistic pathogens including *Enterococcus gallinarum*, *Clostridium innocuum*, and *Clostridium sordellii* were elevated in the CDI group (Figure 7(d,h)).

**Figure 8.**
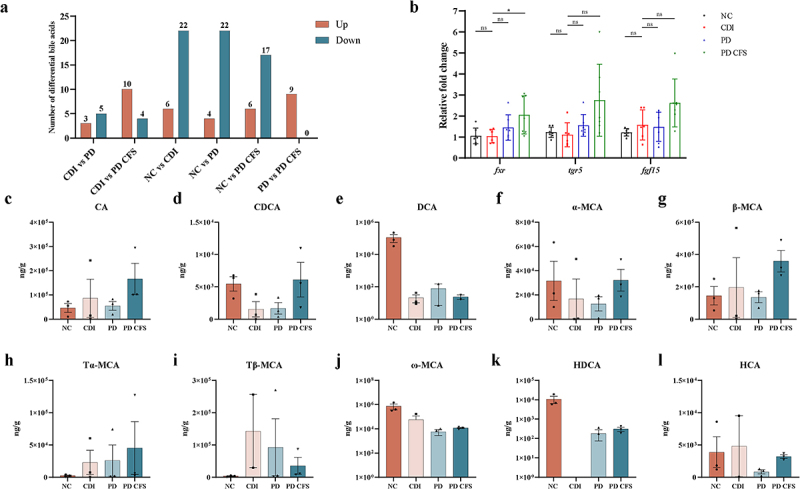
Bile acid metabolic profile is reshaped by *P. distasonis* treatment. (a) Number of upregulated and downregulated bile acids in pairwise intergroup comparison. (b) mRNA expression of bile acid receptors (*n* = 7). (c-l) Relative levels of individual bile acids (*n* = 3). Data are presented as mean ± SEM, analyzed by one-way ANOVA and Bonferroni post-hoc test. *p < 0.05; ns, not significant.CA, Cholic acid; CDCA, Chenodeoxycholic acid; DCA, Deoxycholic acid; LCA, Lithocholic acid; α/β-MCA, α/β-muricholic acid; 7-KLCA, 7-ketolithocholic acid; UDCA, Ursodeoxycholic acid.C.

To further elucidate the metabolic mechanisms underlying these changes, targeted metabolomic profiling of cecal bile acids was conducted. As shown in Figure 8(a), the numbers of differentially expressed bile acids differed among groups. Compared with the CDI group, the number of upregulated bile acids was greater than the number of downregulated bile acids in the PD CFS group; however, the same result was not observed in the PD group. Volcano plot analysis revealed consistent upregulation of HDCA in both treatment groups (Figure S4). HDCA also inhibited *C. difficile* growth, spore formation, biofilm formation, and auto-aggregation in vitro (Figure S5), consistent with the effects observed for DCA. However, HDCA did not significantly inhibit the expression of *C. difficile* virulence genes. Overall, both treatments induced a profound remodeling of the bile acid pool (Figure 8(c-l)). Analysis of colonic bile acid receptor expression further showed that *fxr* expression was elevated in the PD CFS group, while *tgr5* and *fgf15* exhibited increasing trends without statistical significance (Figure 8(b)).

## Discussion

As a core member of the human gut microbiota, *P. distasonis* has been inversely associated with various diseases [14–16]. However, its role in CDI has remained largely unexplored. In this study, we demonstrated that *P. distasonis* alleviates CDI through multiple protective mechanisms. Specifically, it converts CA to DCA in vitro, and the resulting DCA directly inhibits *C. difficile* growth and spore formation. Furthermore, *P. distasonis* treatment modulates the host bile acid pool, restores intestinal barrier integrity, and corrects gut microbiota dysbiosis.

The ability to tolerate gastric acid and bile salts is essential for orally administered probiotics to reach the colon, and auto-aggregation facilitates intestinal colonization. Higher auto-aggregation rates often indicate stronger adhesion to epithelial surfaces, enabling competition with pathogens for attachment sites and maintenance of microbial homeostasis [29]. In this study, all four *P. distasonis* strains exhibited strong tolerance to acid and taurocholate, as well as high auto-aggregation activity. Antibiotic susceptibility testing showed that the strains exhibited tolerance to CDI therapeutic agents (metronidazole, vancomycin, and fidaxomicin), suggesting that *P. distasonis* could be co-administered with antibiotics without compromising treatment efficacy, which represents a key advantage for clinical translation.

The ability of *P. distasonis* to transform bile acids is a key mechanism underlying its inhibition of *C. difficile*. Wang et al. [13] first reported that *P. distasonis* converts primary bile acids such as taurocholic acid into secondary bile acids, including DCA and LCA. Primary bile acids such as CA serve as germinants for *C. difficile* spores, whereas secondary bile acids such as DCA, LCA and UDCA inhibit spore germination, growth and sporulation [9,30]. Our study demonstrated that the supernatant of *P. distasonis* co-cultured with CA inhibited *C. difficile* growth, auto-aggregation, and biofilm formation. The inhibitory effect on sporulation was strain-dependent, which is consistent with Thanissery et al. [9]. Notably, while the supernatant reduced biofilm biomass, spore counts, and toxin gene transcription, it did not consistently reduce toxin protein levels and even upregulated certain biofilm-related genes (e.g. *pilA1*, *pilB1*, *slpA*). This decoupling between phenotype and transcription suggests the involvement of post-transcriptional or translational regulatory mechanisms, as well as strain-dependent differences in responsiveness to secondary bile acids. Toxin expression is regulated by TcdR/TcdC, and sporulation depends on Spo0A and the downstream cascade of σ factors (e.g. SigF, SigG). Secondary bile acids may act indirectly by perturbing these upstream regulatory networks rather than directly targeting toxin or sporulation genes. The upregulation of biofilm-related genes may represent a compensatory stress response under subinhibitory conditions, whereas secondary bile acids may inhibit biofilm maturation by disrupting gene product functions or downstream pathways. Collectively, these findings indicate that transcriptional changes alone are insufficient to accurately predict complex phenotypic outcomes. Future studies should directly measure Spo0A phosphorylation levels, toxin protein secretion, and biofilm extracellular matrix components to identify the precise inhibitory targets of this supernatant.

Previous studies reported that subinhibitory DCA induces *C. difficile* biofilm formation [31]. In contrast, we found that both *P. distasonis* supernatant and exogenous DCA inhibited biofilm formation. This discrepancy may be explained by strain-specific differences in biofilm-forming capacity and bile acid sensitivity, as well as experimental conditions. Specifically, Dubois et al. [31] used 240 µM DCA and observed induction, whereas our inhibitory concentrations were 250–500 µM, suggesting a concentration-dependent switch from induction to inhibition. Additionally, DCA at these concentrations inhibits vegetative growth, directly reducing viable cells available for biofilm formation. Notably, Dubois et al. [31] also reported growth inhibition at ≥600 µM DCA, consistent with our findings. Collectively, our results indicate that DCA regulates *C. difficile* biofilm formation in a concentration- and strain-dependent manner, providing evidence for inhibition at higher subinhibitory concentrations.

Beyond its direct antibacterial activity, *P. distasonis* also mitigates CDI by restoring intestinal barrier function. *C. difficile* toxins disrupt epithelial integrity and increase intestinal permeability, leading to diarrhea and inflammation. Similar to barrier-protective probiotics such as *Lactobacillus plantarum* and *Akkermansia muciniphila* [32–34], *P. distasonis* enhances intestinal barrier function in murine models of aging, insulin resistance, and colitis [35–37]. Our findings extend this evidence to CDI, showing that *P. distasonis* upregulated colonic Muc2 and ZO-1 expression and promoted goblet cell proliferation. Although mRNA expression of barrier-related genes did not reach statistical significance, this discrepancy may reflect temporal dynamics in host response. Transcriptional changes typically occur early after infection, while protein accumulation and tissue remodeling manifest later. Fachi et al. [38] assessed mRNA expression in CDI mice two days post-infection. Thus, the protein-level improvement observed in our study likely represents a cumulative outcome of earlier transcriptional activation, with our sampling point missing the transcriptional peak.

Primary bile acids are steroid compounds synthesized from cholesterol in the liver and are typically secreted into the intestine in conjugated forms, such as tauro/glycocholic acid and tauro/glycochenodeoxycholic acid. Mice primarily secrete bile acids into the small intestine in the form of tauro-α/β-muricholic acid (T-α/β-MCA). After deconjugation, free bile acids (α-MCA and β-MCA) are generated and subsequently modified by gut bacteria in the colon to produce secondary bile acids such as ω-MCA, hyocholic acid, and HDCA [39]. In this study, although distinct secondary bile acids were produced in vitro and in vivo, *P. distasonis* consistently regulated bile acid metabolism in both settings. Under in vitro monoculture conditions lacking complex microbial interactions, *P. distasonis* primarily facilitated the conversion of CA to DCA, suggesting its potential to enhance 7α-dehydroxylation activity. In contrast, in the complex intestinal ecosystem, *P. distasonis* administration markedly elevated HDCA levels in mice.

HDCA is a secondary bile acid whose formation is tightly associated with microbial activity in the gut. Makki et al. [40] reported a strong positive correlation between cecal HDCA levels and the abundance of several bacterial families, including *Lachnospiraceae*, *Prevotellaceae*, *Muribaculaceae*, *Burkholderiaceae*, *Bifidobacteriaceae*, and *Ruminococcaceae*. Members of these families, particularly *Lachnospiraceae*, have been demonstrated to encode 7α-dehydroxylase (baiJ), an enzyme capable of converting the primary bile acid β-MCA into HDCA. In our study, *P. distasonis* treatment significantly enriched *Parabacteroides goldsteinii* and elevated HDCA levels. These findings suggest that *P. distasonis* may remodel the gut microbial community to promote the proliferation of commensal species, including *P. goldsteinii*, thereby indirectly enhancing 7α-dehydroxylation pathways and facilitating HDCA synthesis and accumulation.

Bile acids also function as signaling molecules that activate nuclear and membrane receptors, including farnesoid X receptor (FXR) and Takeda G protein-coupled receptor 5 (TGR5), in the intestine and liver [41]. Activation of these receptors suppresses inflammatory responses by inhibiting NF-κB phosphorylation and nuclear translocation. In this study, *P. distasonis* intervention, particularly its cell-free supernatant, upregulated *fxr* expression in colonic tissues. HDCA has been reported to exert metabolic and anti-inflammatory effects in multiple animal models [42]. Taken together, these findings suggest that *P. distasonis* may contribute to host metabolic and immune homeostasis by promoting HDCA production and modulating the bile acid-FXR signaling axis.

Despite these insights, several limitations should be acknowledged. First, significant species differences exist in bile acid composition, conjugation, and metabolism between mice and humans. The human bile acid pool is dominated by CDCA and DCA, whereas the murine pool is enriched in MCAs due to mouse-specific CYP2C70 expression. Humans primarily conjugate bile acids with glycine, while mice use almost exclusively taurine, affecting solubility, transport, and FXR activation. HDCA has been shown in mouse models to increase the bile acid pool and promote cholesterol metabolism via intestinal FXR inhibition and *cyp7a1* upregulation. However, endogenous HDCA levels in humans are extremely low, and its functional role remains poorly understood. Therefore, caution is warranted when extrapolating our findings to clinical practice, and future validation using organoids or clinical cohorts is recommended. Second, a limitation of this study is the exclusive use of vegetative *C. difficile* cells for murine infection, whereas human infection is naturally initiated by the ingestion of dormant spores. Thus, our findings should be interpreted with caution, and future studies using purified spores are warranted to confirm the observed phenotypes. Third, our findings show that *P. distasonis* intervention correlates with increased HDCA levels and FXR expression in mice, but the precise microbial origin and key metabolic enzymes responsible for HDCA synthesis remain unidentified. Moreover, whether the observed effects depend on FXR receptor activation requires further validation. Future studies should employ gene knockout in key bacterial strains or enzyme-specific functional analyses to elucidate the causal relationship between *P. distasonis* and HDCA biosynthesis. Additionally, functional experiments using receptor-specific antagonists and FXR knockout mice are warranted to verify the critical role of the bile acid-FXR axis in *P. distasonis*-mediated alleviation of CDI.

## Conclusion

In summary, we found that *P. distasonis* converts CA to DCA, which directly inhibits the growth and virulence factor expression of *C. difficile*. It further alleviates CDI by restoring the gut microbiota structure and bile acid homeostasis. This study reveals a key metabolic mechanism by which *P. distasonis* mitigates CDI, providing a novel theoretical basis for developing probiotic intervention strategies targeting bile acid regulation.

## Supplementary Material

Clean Copy of Supplementary Material - QVIR-2026-0069.R2.docx

## Data Availability

The raw 16S rRNA sequencing data generated in this study have been deposited in the NCBI Sequence Read Archive under the BioProject accession number PRJNA1355193 (https://www.ncbi.nlm.nih.gov/bioproject/PRJNA1355193/). The Mass spec-based metabolomics data generated in this study have been deposited in the MetaboLights database (MTBLS13831, https://www.ebi.ac.uk/metabolights/MTBLS13831). The datasets generated during and/or analyzed during the current study are available in the Figshare repository (https://doi.org/10.6084/m9.figshare.31248574).

## Abbreviations

CDI: *Clostridioides difficile* infection
FMT: Fecal microbiota transplantation
CA: Cholic acid
CDCA: Chenodeoxycholic acid
DCA: Deoxycholic acid
LCA: Lithocholic acid
HDCA: Hyodeoxycholic acid
PD: *P. distasonis*
PD CFS: *P. distasonis* cell-free supernatant
