# Supplementary material for: Parabacteroides distasonis alleviates Clostridioides difficile infection in mice while modulating secondary bile acids
Source: Virulence. 2026 Jul 5;17(1):2697574. doi: 10.1080/21505594.2026.2697574 (PMC13348918; doi:10.1080/21505594.2026.2697574)
Supplement: Clean Copy of Supplementary Material - QVIR-2026-0069.R2.docx [file KVIR_A_2697574_SM5229.docx]

| Strain Identifier | Full Name | Origin | Provider |
| --- | --- | --- | --- |
| ATCC 8503 | *Parabacteroides distasonis* | Human | ATCC |
| CGMCC 1.30169 | *Parabacteroides distasonis* | Mouse | Dr. Liu Shuangjiang's Lab |
| 1190003 | *Parabacteroides distasonis* | Human | Dr. Yang Ruifu's Lab |
| F3-A 6JT | *Parabacteroides distasonis* | Human | Dr. Yang Ruifu's Lab |
| ATCC BAA-1382 | *Clostridioides difficile* | Human | ATCC |
| ATCC BAA-1870 | *Clostridioides difficile* | Human | ATCC |
| ATCC 43255 | *Clostridioides difficile* | Human | ATCC |
| ATCC 43598 | *Clostridioides difficile* | Human | ATCC |

**Supplementary Table 1** *C.difficile* and *P. distasonis* strains in this study

**Supplementary Table 2** Primer sequences for PCR amplification of *C. difficile*-specific genes

| Gene | Primer | Sequence (5’ to 3’) |
| --- | --- | --- |
| *tcdA* | tcdA_F | AGATTCCTATATTTACATGACAATAT |
|  | tcdA_R | GTATCAGGCATAAAGTAATATACTTT |
| *tcdB* | tcdB_F | GTGTAGCAATGAAAGTCCAAGTTTACGC |
|  | tcdB_R | CACTTAGCTCTTTGATTGCTGCACCT |
| *spo0A* | spo0A_F | AGCGCAATAAATCTAGGAGCA |
|  | spo0A_R | AGGTTTTGGCTCAACTTGTGT |
| *pilA1* | pilA1_F | TATATGGAGTCTCTGCCTGATAAAG |
|  | pilA1_R | CTTTTTCGACCAGTTTGTCTCCTA |
| *pilB1* | pilB1_F | GGACGTCTAGGTATATTTGAAATGC |
|  | pilB1_R | ATCTGGTCAGCTCCTAAGTCTTTT |
| *slpA* | slpA_F | AATGATAAAGCATTTGTAGTTGGTG |
|  | slpA_R | TATTGGAGTAGCATCTCCATC |
| *fliC* | fliC_F | ATGAGAGTTAATACAAATGTAAGTGC |
|  | fliC_R | CTATCCTAATAATTGTAAAACTCC |
| *fliD* | fliD_F | ATGTCAAGTATAAGTCCAGTAAG |
|  | fliD_R | TTAATTACCTTGTGCTTGTG |
| *cwp84* | cwp84_F | TGGGCAACTGGTGGAAAATA |
|  | cwp84_R | TAGTTGCACCTTGTGCCTCA |
| *16S rRNA* | 16S_F | AGAGTTTGATCCTGGCTCAG |
|  | 16S_R | GGTTACCTTGTTACGACTT |

**Supplementary Table 3** Primer sequences for PCR amplification of mouse target genes

| Gene | Primer | Sequence (5’ to 3’) |
| --- | --- | --- |
| *IL-1β* | IL-1β_F | TGCCACCTTTTGACAGTGATG |
|  | IL-1β_R | AAGGTCCACGGGAAAGACAC |
| *IL-6* | IL-6_F | TTTCCTCTGGTCTTCTGGAGTA |
|  | IL-6_R | CTCTGAAGGACTCTGGCTTTG |
| *IL-10* | IL-10_F | CCCTTTGCTATGGTGTCCTTTC |
|  | IL-10_R | AGGATCTCCCTGGTTTCTCTTC |
| *IL-21* | IL-21_F | GCCAGATCGCCTCCTGATTA |
|  | IL-21_R | CATGCTCACAGTGCCCCTTT |
| *IL-22* | IL-22_F | GTGAGAAGCTAACGTCCATC |
|  | IL-22_R | GTCTACCTCTGGTCTCATGG |
| *TNF-α* | TNF-α_F | GTAGCCCACGTCGTAGCAAA |
|  | TNF-α_R | ACAAGGTACAACCCATCGGC |
| *MUC2* | MUC2_F | GAAGCCAGATCCCGAAACCA |
|  | MUC2_R | GAATCGGTAGACATCGCCGT |
| *ZO-1* | ZO1_F | GCTGCCTCGAACCTCTACTC |
|  | ZO1_R | TTGCTCATAACTTCGCGGGT |
| *Claudin-1* | Claudin-1_F | ACTGTGGATGTCCTGCGTTT |
|  | Claudin-1_R | TCATGCCAATGGTGGACACA |
| *Claudin-3* | Claudin-3_F | TCGAAGGGCAGTTGATTCCC |
|  | Claudin-3_R | ACATGGCTGCTGGACTTGAA |
| *Occludin* | Occludin_F | TTTCAGGTGAATGGGTCACCG |
|  | Occludin_R | TCCAGGCTCCCAAGATAAGC |
| *FXR* | FXR_F | GATGCTGAAGCTTATGCCGGA |
|  | FXR_R | ACTTCTGGGATGGTGGTCCT |
| *TGR5* | TGR5_F | TGTACCCTCAACCCTGGCTA |
|  | TGR5_R | CTGCCCAATGAGATGAGCGA |
| *FGF15* | FGF15_F | CATCTTCATCCAGGCCAAGC |
|  | FGF15_R | TCCATGCTGTCACTCTCCAG |
| *GAPDH* | GAPDH_F | AGAAGGTGGTGAAGCAGGCATC |
|  | GAPDH_R | CGAAGGTGGAAGAGTGGGAGTTG |

**Supplementary Table 4** MICs (mM) of CA, DCA and HDCAagainst *C. difficile* and *P. distasonis* strains

|  | Strains | CA | DCA | HDCA |
| --- | --- | --- | --- | --- |
| *Parabacteroides distasonis* | 1190003 | 256 | 16 | 8 |
|  | CGMCC 1.30169 | 256 | 2 | 4 |
|  | F3-A 6JT | 128 | 16 | 8 |
|  | ATCC 8503 | 256 | 16 | 4 |
| *Clostridioides difficile* | ATCC BAA-1382 | 16 | 1 | 1 |
|  | ATCC BAA-1870 | 16 | 1 | 1 |
|  | ATCC 43255 | 16 | 1 | 1 |
|  | ATCC 43598 | 16 | 1 | 1 |


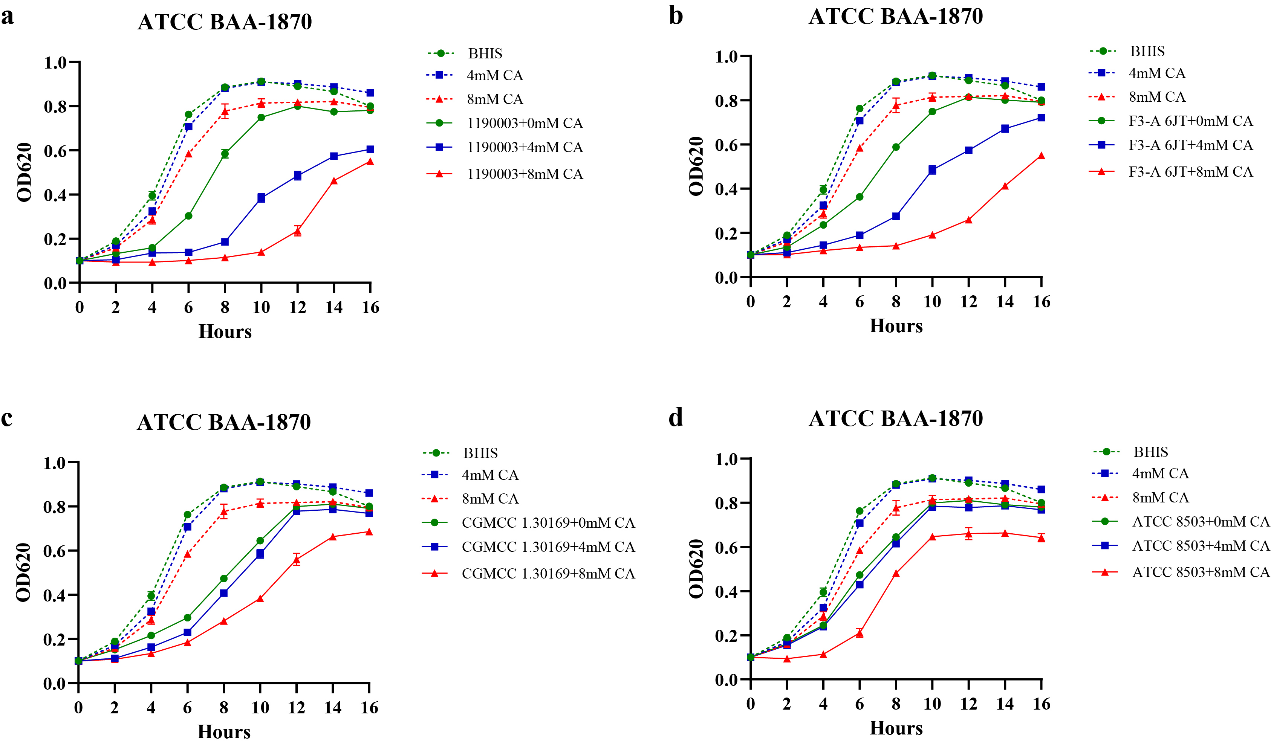


**Supplementary Figure 1.** Growth curves of *C. difficile* in supernatants from *P. distasonis* co-cultured with CA. Growth curves of *C. difficile* ATCC BAA-1870 in supernatants collected from *P. distasonis* strains 1190003 (a), F3-A 6JT (b), CGMCC 1.30169 (c), and ATCC 8503 (d) cultured with 0 mM, 4 mM, or 8 mM CA. Data are presented as mean ± SD.


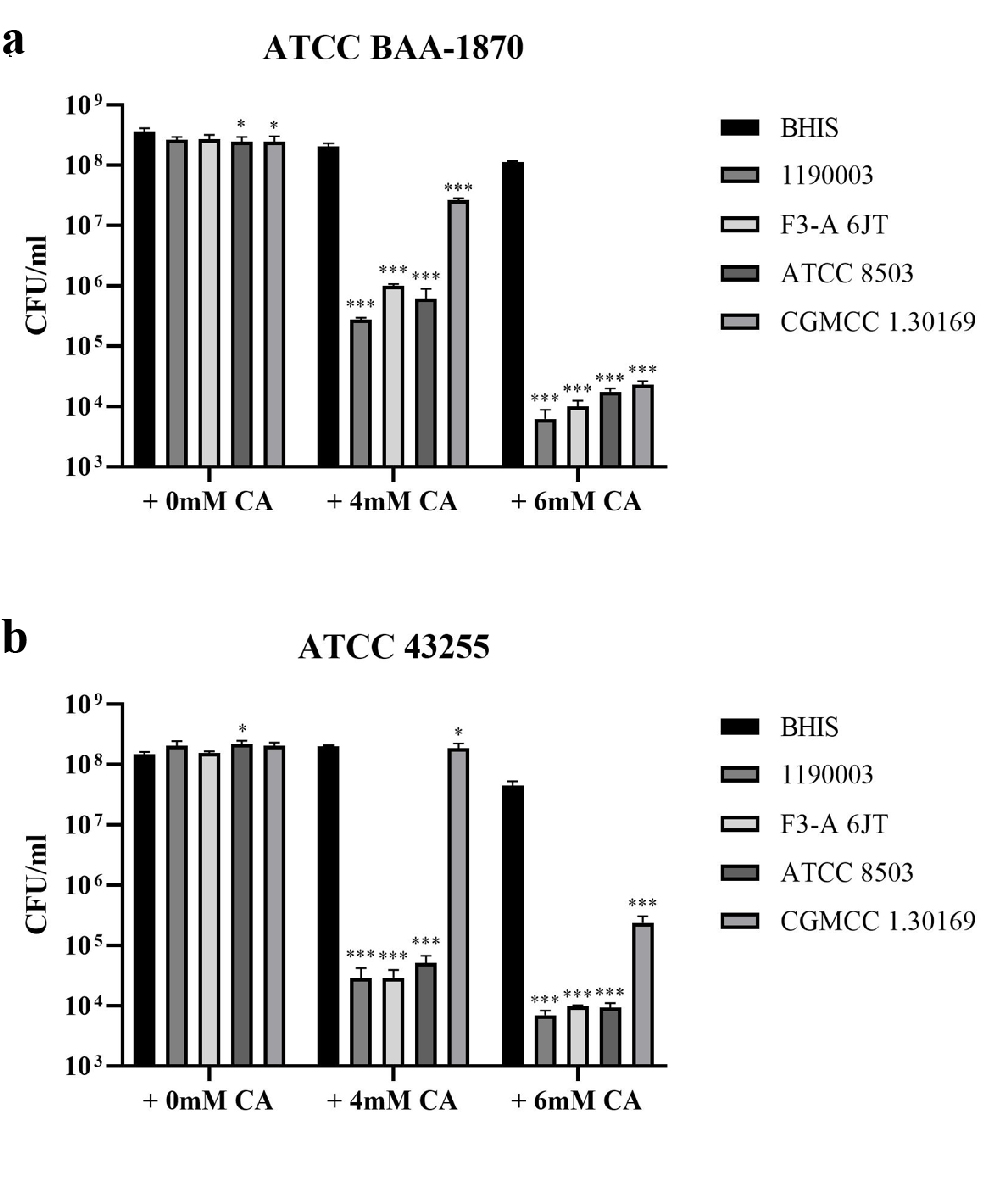


**Supplementary Figure 2.** Colony counts of *C. difficile* after 24 h incubation with supernatants from *P. distasonis* co-cultured with CA. Colony counts of *C. difficile* ATCC BAA-1870 (a) and ATCC 43255 (b) incubated for 24 h in the supernatants from *P. distasonis* strains 1190003, F3-A 6JT, ATCC 8503, and CGMCC 1.30169 cultured with 0 mM, 4 mM, or 6 mM CA. Data are presented as mean ± SD, analyzed by one-way ANOVA and Bonferroni post-hoc test. **P* < 0.05; ***P* < 0.01; ****P* < 0.001.


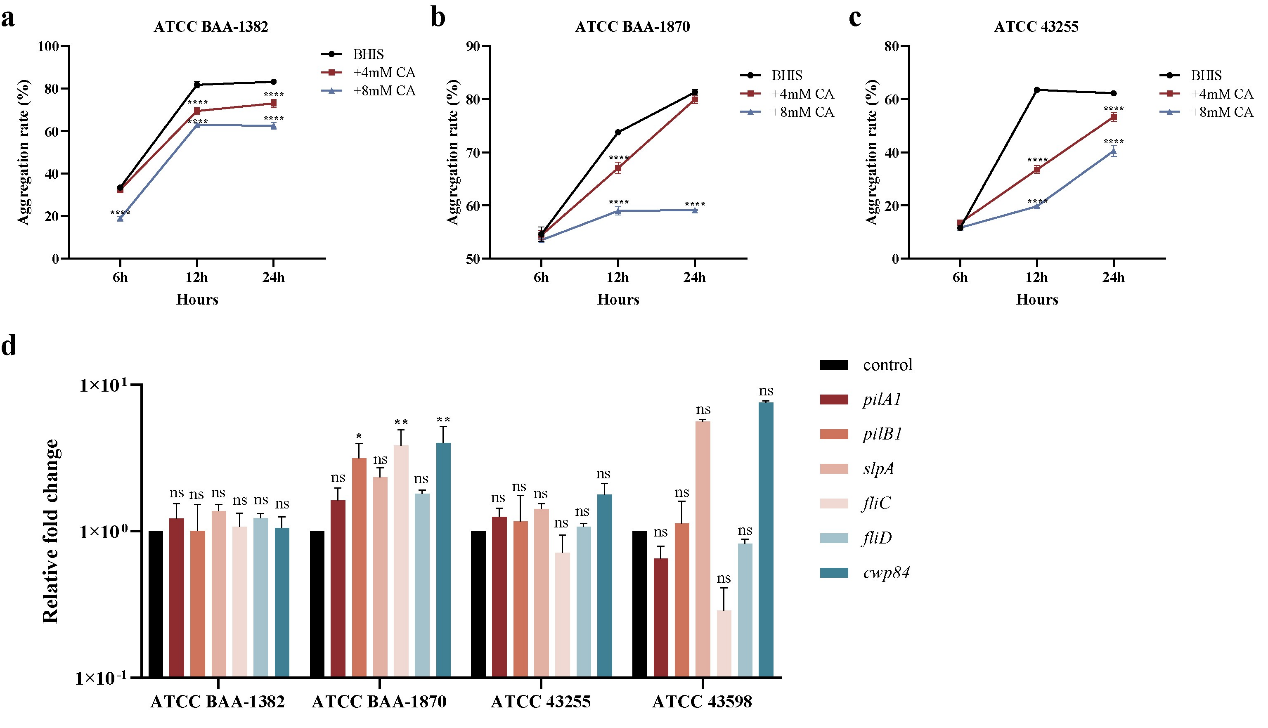


**Supplementary Figure 3.** Auto-aggregation ability and expression of biofilm-related genes in *C. difficile*. Auto-aggregation rate of *C. difficile* ATCC BAA-1382 (a), ATCC BAA-1870 (b), and ATCC 43255 (c). (d) Relative mRNA expression levels of biofilm-associated genes (*pilA1, pilB1, slpA, fliC, fliD, cwp84*) in *C. difficile*. Data are presented as mean ± SD, analyzed by one-way ANOVA and Bonferroni post-hoc test. Two-way ANOVA was used for auto-aggregation data. **P* < 0.05; ***P* < 0.01; ****P* < 0.001; ns, not significant.


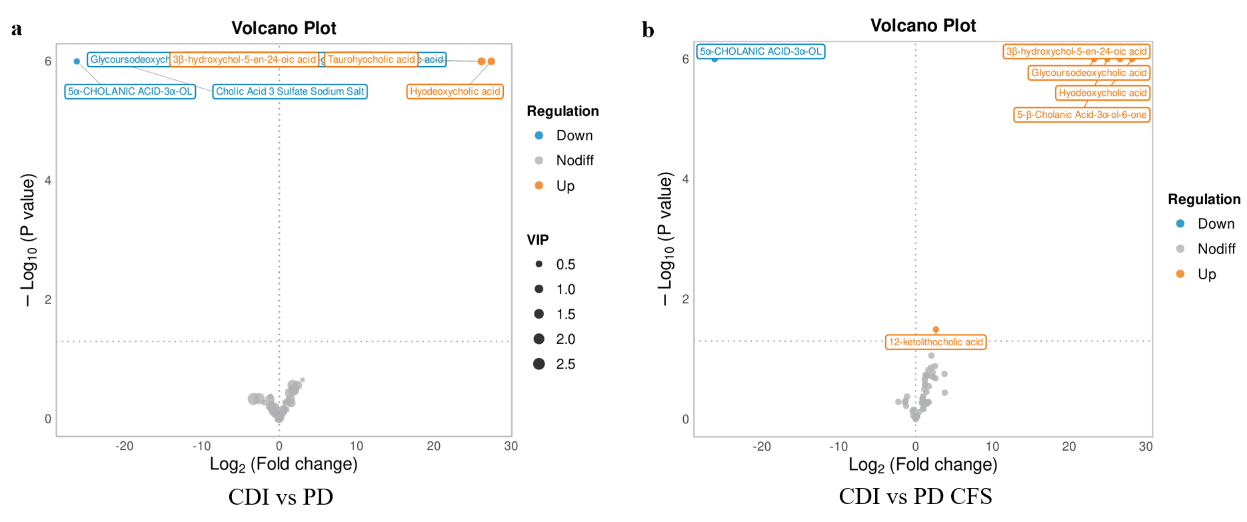


**Supplementary Figure 4.** Volcano plot of differentially abundant metabolites. (a) Comparison between *C. difficile* infection group (CDI) and live *P. distasonis* intervention group (PD), showing results for the PD group. (b) Comparison between CDI group and *P. distasonis* cell-free supernatant intervention group (PD CFS), showing results for the PD CFS group. n=3 per group. HDCA, Hyodeoxycholic acid. Each point represents a metabolite. A larger absolute value on the x-axis reflects a greater fold change in metabolite abundance between the two groups, and a higher value on the y-axis indicates greater statistical significance. Upregulated metabolites are indicated in orange, downregulated metabolites are indicated in blue, and metabolites with no significant difference are indicated in gray.


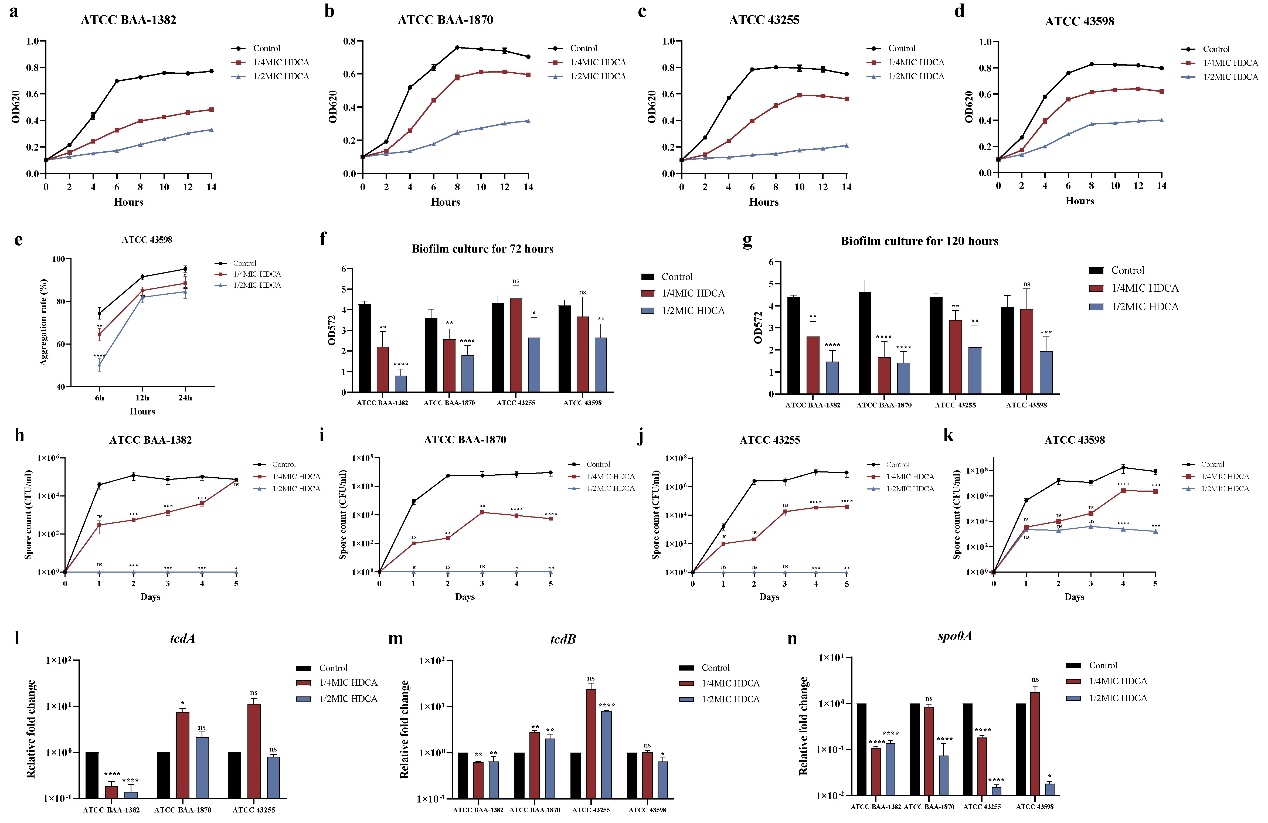


**Supplementary Figure 5.** Effect of subinhibitory concentration of Hyodeoxycholic acid on *C. difficile*. Growth curves of *C. difficile* strains ATCC BAA-1382 (a), ATCC BAA-1870 (b), ATCC 43255 (c), and ATCC 43598 (d). (e) Auto-aggregation rates of *C. difficile* ATCC 43598. Biofilm formation at 72h (f) and 120h (g). Spore counts of *C. difficile* ATCC BAA-1382 (h), ATCC BAA-1870 (i), ATCC 43255 (j) and ATCC 43598 (k). Relative mRNA expression levels of *tcdA* (l), *tcdB* (m) and *spo0A* (n) in *C. difficile*. Data are presented as mean ± SD, analyzed by one-way ANOVA and Bonferroni post-hoc test. Two-way ANOVA was used for auto-aggregation and spore count data. **P* < 0.05; ***P* < 0.01; ****P* < 0.001; ns, not significant.
